# Feasibility of targeted therapies in the adjuvant setting of early breast cancer in men: real-world data from a population-based registry

**DOI:** 10.1007/s00404-024-07405-5

**Published:** 2024-03-12

**Authors:** M. L. Frevert, D. Dannehl, L. Jansen, S. Hermann, H. Schäffler, S. Huwer, W. Janni, I. Juhasz-Böss, A. D. Hartkopf, F.-A. Taran

**Affiliations:** 1 Department of Obstetrics and Gynecology, University Medical Center Freiburg, Faculty of Medicine, University of Freiburg, Hugstetter Str. 55, 79106 Freiburg, Germany; 2 Department of Women’s Health, Tuebingen University Hospital, Tuebingen, Germany; 3 Epidemiological Cancer Registry of Baden-Württemberg, German Cancer Research Center (DKFZ), Heidelberg, Germany; 4 Department of Obstetrics and Gynecology, University Clinic Ulm, Faculty of Medicine, University of Ulm, Ulm, Germany

**Keywords:** Male breast cancer, Systemic therapy, CDK 4/6 inhibitors, monarchE, NATALEE, OlympiA

## Abstract

**Background:**

Following the positive iDFS and OS results of the phase III clinical trials monarchE, NATALEE and OlympiA, new oral anticancer agents (the CDK4/6 inhibitors abemaciclib, ribociclib as well as the PARP inhibitor olaparib) have recently been introduced into the treatment of high-risk early breast cancer (eBC). However, only few male patients were included in these trials (0.4%, 0.6% and 0.3%, respectively). The objective of this real-world analysis was to determine the proportion of male patients with eBC fulfilling the clinical high-risk criteria of above-mentioned trials.

**Patients and methods:**

We conducted a data inquiry and analysis with the Cancer Registry of Baden-Württemberg of men with breast cancer diagnosed between January 1, 2015 and December 31, 2021. Men with eBC were identified and the number of patients at clinical high-risk according to the inclusion criteria of monarchE, NATALEE and OlympiA was assessed.

**Results:**

Of 397 men with eBC, 354 (89.1%) had a HR + /Her2− and 4 (1.0%) a triple-negative subtype. 84 patients (21.2%) met the clinical high-risk criteria according to the monarchE, 189 (47.6%) those according to the NATALEE and 50 (12.6%) those according to the OlympiA trial.

**Conclusion:**

In a large real-world sample, more men with eBC are at clinical high risk according to the inclusion criteria of monarchE, NATALEE and OlympiA than would be expected in women. This is most likely due to more advanced stages at initial diagnosis in men. To evaluate whether CDK4/6 and PARP inhibitors improve prognosis also in men should be the topic of future real- world analyses.

## What does this study adds to the clinical work

**Table:** 

Approximately 1 in 5 men with early breast cancer would meet the clinical high-risk criteria as defined in the monarchE, half the patients those as defined in the NATALEE and 1 in 9 those as defined in the OlympiA trial. We are bound to also see many male patients for extended adjuvant treatment in our chemotherapy outpatient clinics; whether CDK4/6 and PARP inhibitors improve the prognosis in men with eBC must be the topic of future analyses.	

## Introduction

The past couple of years have shown that advances made in the palliative personalized treatment of metastatic breast cancer can also be used in the curative treatment of high-risk early breast cancer (eBC) to prevent or delay the onset of distant metastatic disease. In the monarchE and NATALEE study, patients with hormone receptor positive (HR +)/Her2− eBC at high risk of recurrence received a cyclin-dependent kinase 4/6 (CDK4/6) inhibitor (abemaciclib and ribociclib, respectively) in combination with endocrine therapy [1, 2], while in the OlympiA trial, patients with germline BRCA 1 or BRCA 2 mutations at high clinical risk of relapse received the poly ADP ribose polymerase (PARP) inhibitor olaparib after (neo)adjuvant chemotherapy [3].

In the monarchE trial, 2 years of abemaciclib (2 × 150 mg/day) in combination with standard endocrine therapy significantly improved invasive disease-free survival (iDFS) (*P* = 0.01; hazard ratio (HR), 0.75; 95% confidence interval (CI) 0.60–0.93), leading to the approval of abemaciclib by the European Medicine Agency (EMA). Similarly, in the NATALEE trial, 36 months of ribociclib (1 × 400 mg/day) in combination with an aromatase inhibitor also demonstrated significantly longer iDFS than sole endocrine therapy (HR 0.75; 95% CI 0.62–0.91, *P* = 0.0014). The OlympiA trial included Her2−negative (Her2−) eBC patients harboring a germline BRCA1- or BRCA2 mutation who had previously been treated with an anthracycline and a taxane in the (neo)adjuvant setting [4]. Here, 12 months of treatment with olaparib (2 × 300 mg/day) led to an improved iDFS (HR 0.58; 99.5% CI 0.41–0.82; *P* < 0.0001) [3] and overall survival (OS) (HR 0.68; 98.5% CI 0.47–0.97; *P* = 0.009) compared to placebo [5].

Of all breast cancers diagnosed each year, 0.5–1% are diagnosed in men [6, 7]. The three studies mentioned above also included men, albeit only very few: 36 men (0.6%) in monarchE, 20 men (0.4%) in NATALEE and 6 men (0.3%) in OlympiA.

Baden-Württemberg is the third largest federal state in Germany with a population of 11 million inhabitants, representing approximately 13% of the total German population [8]. From 2009 onwards, a legal obligation to report all pre-malignant and malignant neoplasms to the Cancer Registry of Baden-Württemberg was gradually introduced. Since November 2011, reporting of cancer diagnosis, treatment and progression has been mandatory in Baden-Württemberg [9].

We used the Cancer Registry of Baden-Württemberg to identify characteristics such as age, TNM stage, hormone receptor status, and treatment information of male breast cancer patients. The objective of this registry study was to determine the number of men with eBC who met the clinical high-risk criteria of the three above-mentioned trials in the real-world.

## Materials and methods

In collaboration with the Baden-Württemberg Cancer Registry, we selected and analyzed population-based cancer registry data. Data from men diagnosed with breast cancer for the first time between January 1, 2015 and December 31, 2021 were included in this retrospective analysis.

In our study, the investigators did not have direct access to individual patient data—the Baden-Württemberg Cancer Registry provided the previously aggregated results. Therefore, according to German law, neither ethics committee approval nor informed patient consent was required to conduct our analysis. All patient data were anonymized by the cancer registry. In practice, this meant that any information that could be used to identify an individual patient or a specific hospital was removed by the cancer registry to ensure data protection.

As a consequence, age was only available as a 5-years category. Similarly, certain tumor characteristics were not available to us as they were not collected. These include information on biological subtype, genomic risk profiling (Oncotype Dx, Prosigna PAM 50, Endopredict or Mammaprint), Ki-67 proliferation index and the BRCA1 and BRCA2 germline mutational status.

Exclusion criteria were distant metastatic disease, carcinoma in situ and missing data, be it on TNM stage (neither pathological nor clinical stage had been reported; either clinical TNM and/or pathological TNM stage sufficed for inclusion) or hormone receptor status. Tumors were considered HR + if expression of the estrogen receptor (ER) and/or progesterone receptor (PR) had been reported as positive to the registry.

Patients were then further screened according to the high-risk criteria of monarchE, NATALEE and OlympiA (Table 1). TNM stages were reported according to the pathological criteria, wherever possible. As information on pathological TNM stage was often missing after neoadjuvant chemotherapy had been carried out, either the clinical or the pathological stage was used (depending on which was more advanced or available).

**Table 1:** High risk criteria according to monarchE, NATALEE and OlympiA

monarchE	NATALEE	OlympiA
HR + /Her2−	HR + /Her2−	TNBC or HR + /Her2−
Pathologic lymph node involvement and 1 of the following ≥ 4 positive axillary lymph nodes Tumor size of ≥ 5cm Histologic grade 3 (G3) Centrally assessed Ki-67 ≥ 20%	Anatomical Stage III Anatomical Stage IIB Anatomical Stage IIA with either N1 Or N0 if Histologic grade 3 (G3) Histologic grade 2 (G2) if Ki-67 ≥ 20% Oncotype DX RS ≥ 26 Prosigna PAM 50 high risk MammaPrint high risk EndoPredict high risk	TNBC with non-pCR after neoadjuvant chemotherapy TNBC treated with primary surgery and displaying pathologic lymph node involvement or tumor size ≥ 20 mm HR + /Her2− with non-pCR after neoadjuvant chemotherapy and CPS-EG score ≥ 3 HR + /Her2− treated with primary surgery displaying ≥ 4 pathological lymph nodes

In the monarchE trial, HR + /Her2− lymph-node-positive eBC and at least one of the following characteristics indicating a higher risk of recurrence were considered high risk: 4 or more positive axillary lymph nodes, tumor size of at least 5 cm, grade 3 tumors or centrally assessed Ki-67 ≥ 20%. In our analysis, Ki-67 was not taken into account as a) this marker was not available for us for analysis and b) when abemaciclib was approved for adjuvant therapy of eBC by the European Medical Agency (EMA), the Ki-67 was not considered.

For risk assessment according to the NATALEE trial, HR + /Her2−negative eBC, cases with an Anatomic Stage Group IIB-III and a subset of Stage IIA cases (N0 with G3 or N1) were included.

The clinical high risk criteria according to the OlympiA trial were as follows: (1) patients with triple negative eBC who had either received neoadjuvant chemotherapy and did not show a pathological complete response (pCR) or (2) patients after primary surgery with pathologic lymph node involvement or a tumor size of at least 20 mm. Patients with HR + /Her2− eBC who underwent neoadjuvant chemotherapy also had to display non-pCR, but they additionally needed to exhibit a CPS-EG score of at least 3. The latter is based on pre-treatment clinical (CS) and post-treatment pathological stage (PS), the estrogen receptor status (E) and grade (G) [10–12]. Patients with HR + /Her2− eBC who had not received neoadjuvant chemotherapy had to have at least four pathologically involved lymph nodes.

Data processing and statistical analyses were performed using Jupyter Notebook (Version 6.3.0, Project Jupyter, open-access and community developed) on Anaconda (Version 3.0, Anaconda Inc., Austin, TX, USA), with the Python extension packages pandas (Version 1.4.1, open-access and community developed) and numeric Python (Version 1.22.2, open-access and community developed).

## Results

**Fig. 1:**
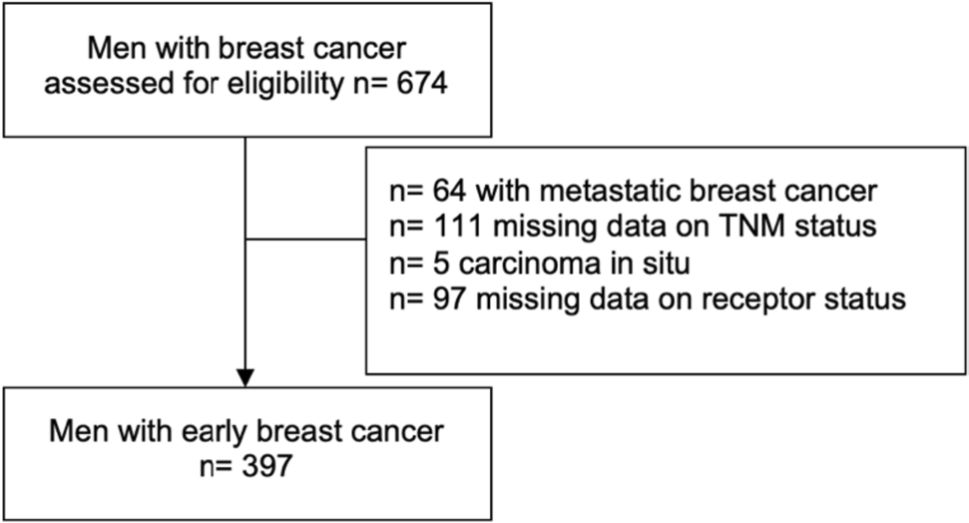
Selection of patients with eBC: The database included 674 male breast cancer patients. 64 patients with metastatic breast cancer, 111 patients with missing data on TNM stage (no clinical or pathological information had been reported to the registry), 5 patients with carcinoma in situ and 97 patients with missing data on receptor status were excluded. Hence, the final study population consisted of 397 patients with early breast cancer

Between January 1, 2015 and December 31, 2021, 674 men with breast cancer were reported to the Cancer Registry of Baden-Württemberg (as depicted in Fig. 1 below), 397 of whom were patients with early breast cancer: 354 (89.2%) had a HR + /Her2−, 39 (9.8%) a Her2 + , and 4 (1.0%) a triple-negative tumor subtype. The mean age at diagnosis was 72.5 ± 12.5 years (Table 2).

**Table 2:** Characteristics of the overall male eBC population and the patients at clinical high risk according to the NATALEE, monarchE and OlympiA trials, with percentages referring to the respective study population in parentheses and T-stage and N-stage referring to the pathological stage

	Overall	High-risk according to monarchE	High-risk according to NATALEE	High-risk according to OlympiA
	397 (100%)	84 (100%)	189 (100%)	50 (100%)
Age (Mean ± SD)	72.5 ± 12.5	73.3 ± 10.3	72.8 ± 11.1	72.8 ± 11.1
Grading				
1	33 (8.3%)	2 (2.4%)	5 (2.6%)	2 (4%)
2	241 (60.7%)	33 (39.3%)	111(58.7%)	31 (62%)
3	116 (29.2%)	48 (57.1%)	71 (37.6%)	16 (32%)
n/a	7 (1.8%)	1 (1.2%)	2 (1.1%)	1 (2%)
T-stage				
0^*^	1 (0.3%)	0	0	0
1	136 (34.3%)	16 (19.0%)	45 (23.8%)	9 (18%)
2	153 (38.5%)	40 (47.6%)	89 (47.1%)	24 (48%)
3	7 (1.8%)	4 (4.8%)	5 (2.6%)	2 (4%)
4	51 (12.8%)	23 (27.4%)	41 (21.7%)	15 (30%)
n/a	49 (12.3%)	1 (1.2%)	9 (4.8%)	0
N-stage				
0	199 (50.1%)	0	30 (15.9%)	1 (2%)
1	134 (33.8%)	36 (42.9%)	111 (58.7%)	1 (2%)
2	43 (10.8%)	37 (44.0%)	37 (19.6%)	37 (74%)
3	14 (3.5%)	11 (13.1%)	11 (5.8%)	11 (22%)
n/a	7 (1.8%)	0	0	0
ER status				
+	393 (99.0%)	84 (100%)	189 (100%)	48 (96%)
–	4 (1.0%)	0	0	2 (4%)
PR status				
+	362 (91.2%)	81 (96.4%)	175 (92.6%)	47 (94%)
–	35 (8.8%)	3 (3.6%)	14 (7.4%)	3 (6%)
Her2 status				
+	39 (9.8%)	0	0	0
–	358 (90.2%)	84 (100%)	189 (100%)	50 (100%)
Chemotherapy				
Neoadjuvant	13 (3.3%)	1 (1.2%)	9 (4.8%)	0
Adjuvant	68 (17.1%)	31 (36.9%)	43 (22.8%)	21 (42%)
None/not reported	318 (80.1%)	52 (61.9%)	137(72.5%)	29 (58%)
Biology				
HR + /Her2−	354 (89.2%)	84 (100%)	189 (100%)	48 (96%)
HR + /Her2 +	39 (9.8%)	0	0	0
TNBC	4 (1.0%)	0	0	2 (4%)

*SD* standard deviation, *n/a* not available, *ER* estrogen receptor, *PR* progesterone receptor, *Her2* human epidermal growth factor 2, *HR* hormone receptor, *TNBC* triple negative breast cancer

^*^One patient in the overall analysis was clinically stage T1, but pathologically stage T0. For reasons of completeness, we kept him in the analysis

The most common histological grading in our cohort was G2 (60.7%), 72.8% of tumors were stage T1/T2 (289 in total) and 191 patients had positive nodes (48.1%). 80.1% of patients did not undergo chemotherapy (or such treatment was not reported to the registry) (79.8% with HR + and 59% with Her2− eBC). 17.1% received adjuvant and 3.3% received neoadjuvant chemotherapy.

**Fig. 2:**
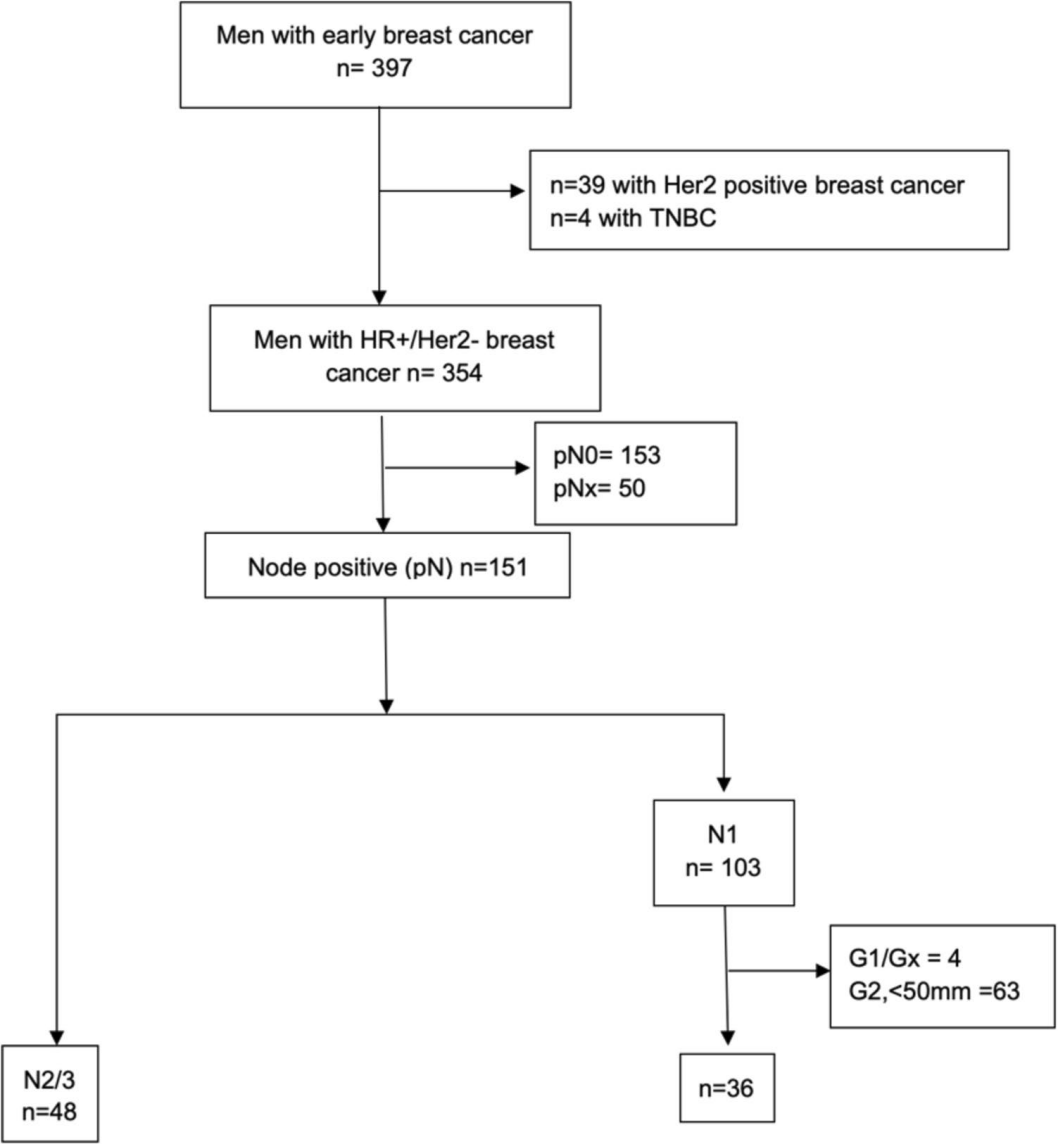
Selection of patients at clinical high risk according to the monarchE trial: Of the 397 patients with localized disease, 354 had a HR + /Her2− breast cancer subtype. 172 of those displayed involved lymph nodes: 48 patients with N2/3 (≥ 4 involved lymph nodes) and 124 patients with N1 (1–3 involved lymph nodes). Of this latter category, 67 had a grading of 2 or lower (G1/G2/Gx) and/or a tumor size of < 5 cm, leaving 36 patients with N1/G3 and/or tumor size ≥ 5 cm to be eligible for inclusion. All in all, 84 patients met the monarchE trial clinical high-risk criteria

As displayed in Fig. 2, 84 patients (21.2%) were at clinical high risk according to the monarchE study. The mean age of these patients was 73.3 ± 10.3 years. 56/84 patients (66.7%) had a tumor stage of T1/2 and 48/84 (57.1%) had at least 4 positive nodes (Table 2). Only 1 patient (1.2%) had received neoadjuvant chemotherapy, whereas 31 patients (36.9%) had received adjuvant chemotherapy.

**Fig. 3:**
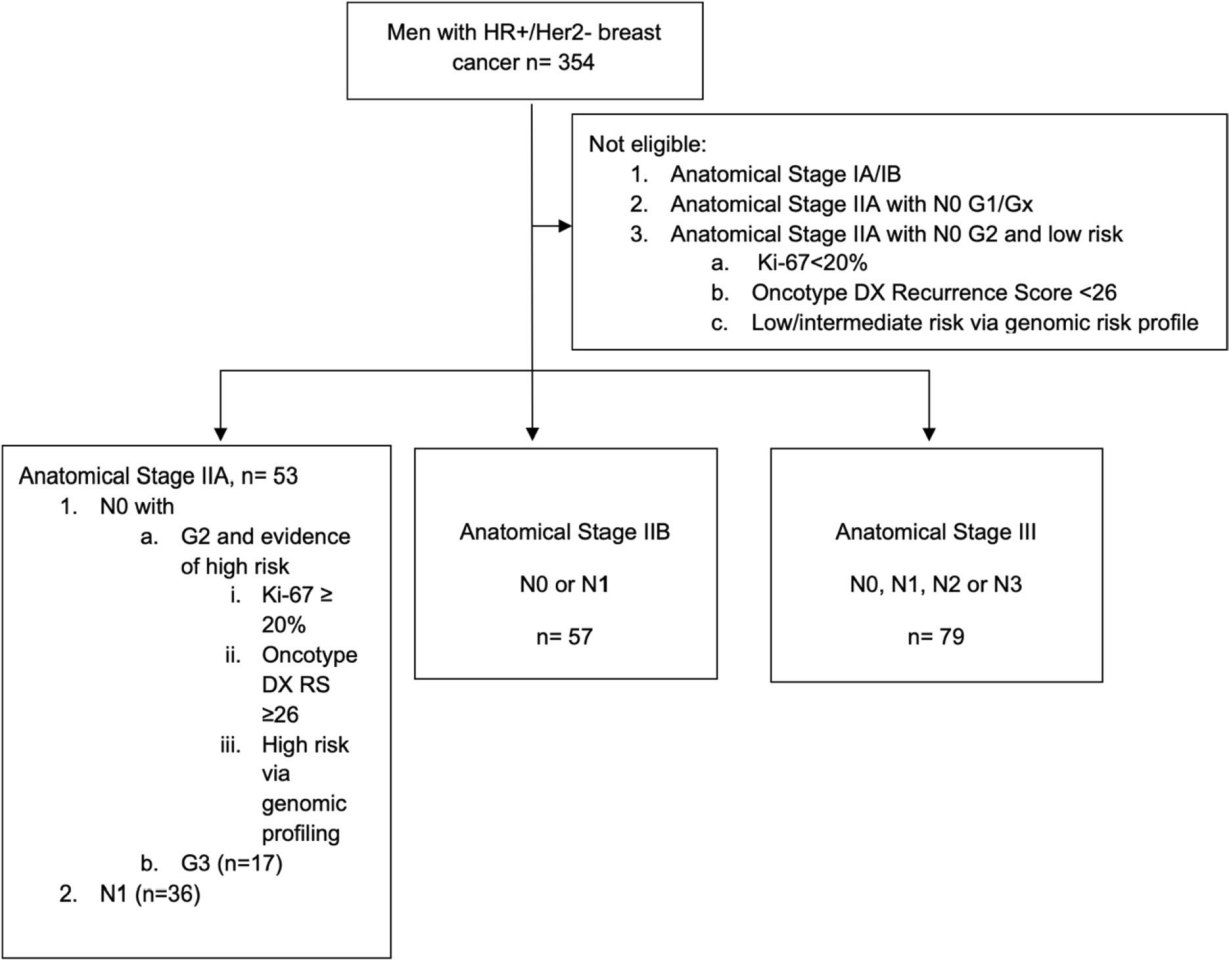
Selection of patients at clinical high risk according to the NATALEE trial: Of the 354 patients with a HR + /Her2− breast cancer subtype, 53 had Anatomic Stage Group IIA disease (36 with positive lymph nodes, 17 with no lymph node involvement but G3 grading as a marker of high risk), 57 with Anatomic Stage Group IIB disease, and 79 had Anatomic Stage Group III disease. All in all, a total of 189 patients met the clinical high-risk criteria of the NATALEE trial

As shown in Fig. 3, 189 patients (47.6%) were at clinical high risk according to the NATALEE trial with a mean age of 72.8 ± 11.1 years. 28% of patients (53/189) had Anatomic Stage Group IIA disease, 30.2% (57/189) Anatomic Stage Group IIB and 41.8% (79/189) Anatomic Stage Group III disease. 30/189 patients (15.8%) displayed no lymph node involvement. Nine patients received neoadjuvant chemotherapy (4.76%) and 43/189 patients (22.8%) received adjuvant chemotherapy (Table 2).

**Fig. 4:**
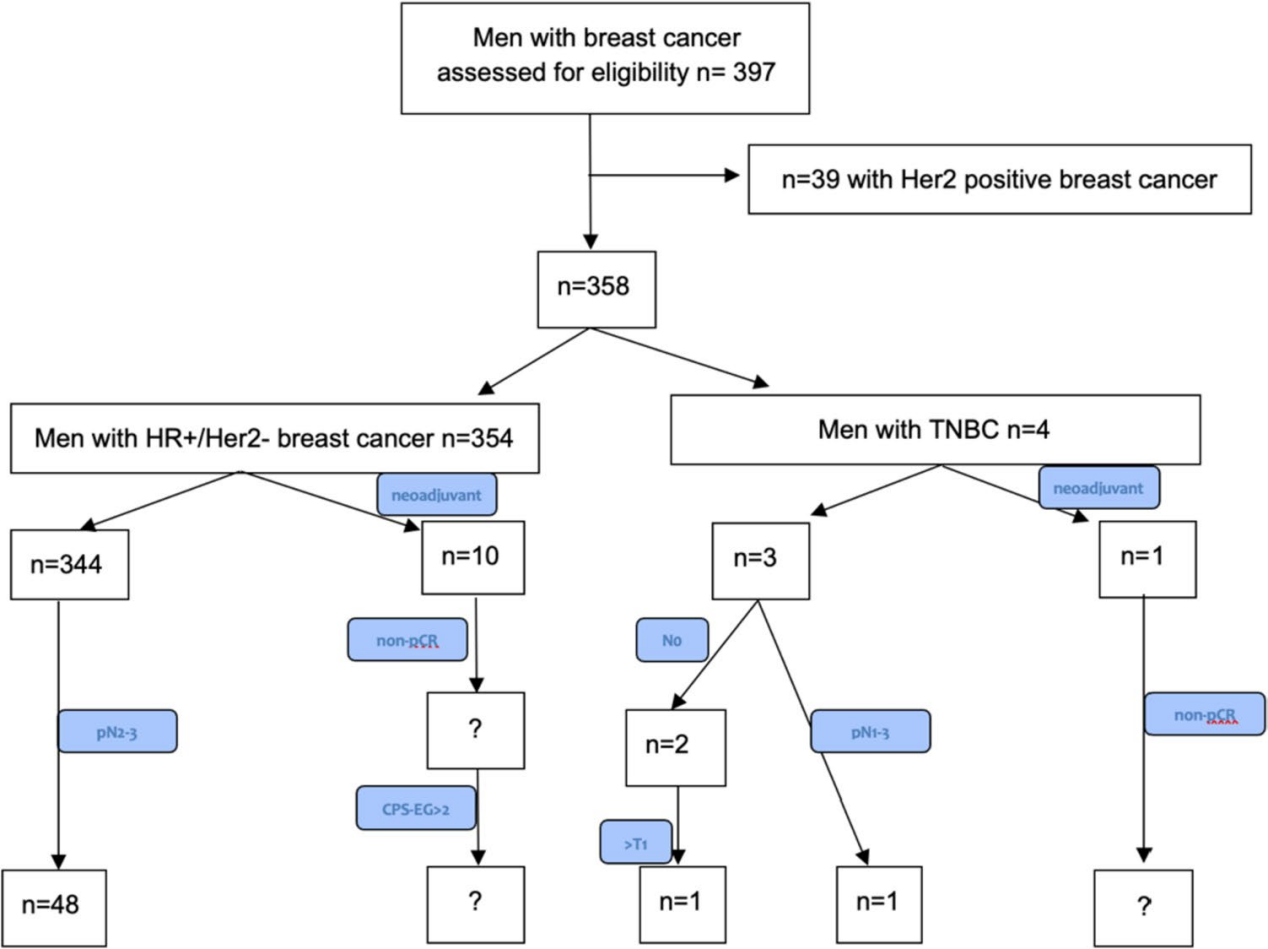
Selection of patients at clinical high risk according to the OlympiA trial: Of 397 patients assessed for eligibility, 354 were HR + /Her2− and 4 patients were triple-negative. 53 patients in the HR + /Her2− and 1 patient in the TNBC group received adjuvant chemotherapy; 10 patients of the HR + /Her2− and 1 patient of the TNBC group received neoadjuvant chemotherapy. Of the 344 HR + /Her2− patients who were not treated with neoadjuvant chemotherapy (or where no neoadjuvant chemotherapy had been reported to the registry), 48 patients (96%) showed pathological involvement of at least 4 lymph nodes (pN2-3) and 20 of these 48 (41.7%) received adjuvant chemotherapy. Of the 3 patients with TNBC who were not treated with neoadjuvant chemotherapy (or where no neoadjuvant chemotherapy had been reported to the registry), one patient displayed pathological lymph node involvement (pN1-3) and received adjuvant chemotherapy and 1 patient was node negative with a tumor size greater than 20 mm (> T1)

Figure 4 displays the 50 patients (12.6%) fulfilling the clinical high-risk criteria according to the OlympiA trial. 48/50 (96.0%) were HR + /Her2− and 2/50 (4.0%) were HR−/Her2−. The mean age was 72.8 ± 11.1 years (Table 2). Of the 2 triple negative patients, 1 received adjuvant chemotherapy (50.0%) and of the 48 patients with HR + /Her2− eBC 20 (41.7%) received adjuvant chemotherapy. Due to the lack of information on pathological complete response (pCR) in the data, patients who had received neoadjuvant chemotherapy could not be included in the analysis.

The question marks (?) in Fig. 4 are used to illustrate that information on pCR and, in the case of the HR + /Her2− tumors, CPS-EG Score could not be derived from the available data and that these patients had to be excluded from our analysis due to missing data on pCR.

## Discussion

In this analysis, we were able to show that 21.2% and 47.6% of male patients with HR + /Her2− eBC met the high-risk criteria of the monarchE and NATALEE trial, respectively, and that 12.6% of male patients with eBC met the high-risk clinicopathological criteria of the OlympiA study. This is a much higher percentage of patients than has been reported in the real-world data for women [13, 14], which might be due to the fact that men tend to be diagnosed at a more advanced stage [15–18].

Considering that in our OlympiA analysis, only patients with no record of chemotherapy or adjuvant chemotherapy were included as information about non-pCR and CPS-EG scores for patients receiving neoadjuvant chemotherapy was missing or could not be calculated based on the information at hand, we believe that we have even underestimated the number of patients who would meet the high-risk clinicopathological criteria used for inclusion in the trial. Similarly, in our analysis in analogy to the high-risk criteria of the NATALEE trial, we had to exclude 112 patients with an anatomical stage IIA with a G2 grading due to missing data on Ki-67 and genomic profiling tests. Hence, we believe that, here too, the number of patients eligible for treatment with ribociclib in the real world is higher than the numbers we depict.

On the other hand, we were able to show that the patient cohort characterized in this analysis differs from that of the original studies as fewer patients than in the original trials seem to have received chemotherapy: 62% of all patients meeting the high-risk criteria of monarchE, did not undergo chemotherapy (or no information about a chemotherapy conducted had been reported to the registry) versus 4.6% in the original study (where 95.4% received chemotherapy); 72% of all patients meeting the high-risk criteria of NATALEE did not receive chemotherapy (or no information about a chemotherapy conducted had been reported to the registry) versus 12% in the original study (where 88% received chemotherapy); 58% of patients meeting the high-risk criteria of the OlympiA trial did not receive chemotherapy (or no information about a chemotherapy conducted had been reported to the registry) versus 0% in the original study (where 100% received chemotherapy, be it adjuvant or neoadjuvant as it was a prerequisite for being enrolled in the trial). It needs to be mentioned, however, that the mean age of the patients in the original trials was well below that of our cohort: in monarchE, patients’ median age lay at 51.0 years (and only 15.1% of patients were ≥ 65 years old) [1, 19] in NATALEE at 52 years [20] and in OlympiA at 42 years [3], whereas in the cohort here analyzed the mean age lay at 73.3 ± 10.3 years for the patients meeting the clinical high-risk criteria of monarchE and at 72.8 ± 11.1 years for patients meeting those of NATALEE and OlympiA. This is in line with research showing that men are generally older than women at diagnosis [21–23]. Therefore, one possible explanation for the omission of (neo)adjuvant chemotherapy in many patients in our cohort despite a clear indication based on clinicopathological features is age. Other possible explanations for the omission of chemotherapy are the underreporting of chemotherapy treatment to the registry, patient choice, comorbidities or, in the case of HR + /Her2− cancers, prognostic markers such as multigene expression arrays (the results of which were not available to us), where in cases of low risk, chemotherapy can safely be omitted on an individual basis [24–28]. It should also be emphasized that despite male breast cancer being such a rare disease with an absolute incidence case number between 690 and 770 [29], its treatment in Germany unfortunately is not centralized. This means that patients may forego optimal treatment (such as chemotherapy, for example), because they are not seen in certified cancer centers.

Deleterious BRCA2 mutations have been observed in 4 to up to 14% of male breast cancers [27–31], whereas BRCA 1 mutations are rare, except in individuals of Ashkenazi Jewish ethnicity [30, 31]. Recently, a phenotype suggesting more aggressive tumor behavior has been associated with BRCA2 male breast cancer [32, 33]. Thus, it is therefore likely that proportionately more men than women with eBC (where approximately 5% carry pathogenic germline variants in BRCA1 and 2 [34, 35]) will benefit from the treatment with olaparib.

By nature, our retrospective analysis of real-world data from a population-based registry has certain limitations that warrant mention: First and foremost is missing data- important information about pathological complete response, CPS-EG status, tumor biology, Ki-67, and gene expression tests (not to mention genetic mutations) was not available to us for analysis. Similarly, information on treatments performed may not have been reported fully to the registry, so that it may very well be the case that we underestimated the number of patients treated with chemotherapy as this may have been underreported. We are aware that these pieces of information are indispensable when analysing male early breast cancer, especially when reviewing whether a certain patient population meets the high-risk characteristics of above-mentioned studies. However, since male breast cancer is relatively rare and, as already mentioned above, breast cancer treatment in Germany is not centralized (so that no central database with claim to completeness about all the important data points exists), we believe that this substantial real-world sample from a population-based registry grants us invaluable insights about a large male eBC patient collective and possible adjuvant treatment options and that the clinical significance is high despite the limitations of missing data.

In our cohort, all patients who fulfilled the high-risk criteria for monarchE, also met those of the NATALEE study. All patients with a HR + /Her2− eBC who fulfilled the clinicopathological high risk criteria for OlympiA, also fulfilled those of the monarchE und NATALEE trials- an overlap of 100%. We thus eagerly await the further follow-up analyses of the trials, including further specifications on adverse events especially in the age group ≥ 70 years, in order to better counsel male patients on which personalized adjuvant therapy would be optimal for each individual.

As the incidence of male breast cancer has been steadily increasing over the past decades [7, 36, 37] and the average time to diagnosis still lies at 6–10 months after observation of first symptoms [38] (mainly attributed to a lack of knowledge, public education and embarrassment [39]), we are bound to see more and more male patients with eBC who are eligible for adjuvant therapy with a CDK4/6 or PARP inhibitor.

## Conclusion

This real-world analysis shows that approximately 1 in 5 men with eBC would meet the clinical high-risk criteria as defined in the monarchE, half the patients those as defined in the NATALEE and 1 in 9 those as defined in the OlympiA trial. Whether CDK4/6 and PARP inhibitors improve the prognosis in men with eBC must be the topic of future analyses. As we have shown, plenty of male patients exist for these treatment options.

## Data Availability

The datasets generated during and/or analysed during the current study are not publicly available due to the legal obligation to protect sensitive health care data of patients in Germany, but are available from the corresponding author on reasonable request.
